# Data related to the sinter structure analysis of titanium structures fabricated via binder jetting additive manufacturing

**DOI:** 10.1016/j.dib.2018.08.135

**Published:** 2018-08-31

**Authors:** Evan Wheat, Mihaela Vlasea, James Hinebaugh, Craig Metcalfe

**Affiliations:** a Multi-Scale Additive Manufacturing Laboratory, Department of Mechanical and Mechatronics Engineering, University of Waterloo, Waterloo, ON N2L 3G1, Canada; b Expanse Microtechnologies Inc., Toronto, Ontario M4R 2H8, Canada

## Abstract

The adoption of metal binder jetting additive manufacturing (AM) for functional parts relies on a deep understanding between the materials, the design aspects, the additive manufacturing process and sintering. This work focuses on the relationship between sintering theory and process outcomes. The data included in this article provides additional supporting information on the authors’ recent publication (Wheat et al., 2018 [1]) on the sinter structure analysis of commercially pure titanium parts manufactured using powder bed binder jetting additive manufacturing. For this work, commercially pure titanium was deployed to study the effect of powder size distributions on green and sintered part qualities (bulk density, relative density, particle size, pore size, sinter neck size). This manuscript includes the overall computed tomography visualization methods and results for the green and sintered samples using uni- and bi-modal powders. Moreover, the effective particle and pore size for the different batches of powder are presented.

**Specifications Table**

**Table:** 

Subject area	Engineering, Materials Science
More specific subject area	Additive Manufacturing
Type of data	Figures
How data was acquired	Design of Experiments, X-ray computed tomography (CT), analytics on CT datasets
Data format	Analyzed
Experimental factors	The samples were manufactured using multiple unimodal and bimodal powder blends using binder jetting additive manufacturing and sintered to study the sinter structure.
Experimental features	For this work, all titanium powders used were plasma atomized, Grade 1 commercially pure (CP). Three stock powder size ranges were purchased for the production of samples. Three size distributions, 0–45 µm (●○○), 45–106 µm (○●○), and 106–150 µm (○○●) size distribution. Two mono-modal powders (Types B ○○● and C ○●○), as well as three bimodal powders (Types A ○●●, D ●○● and E ●●○) were used in the production of samples. The three bi-modal powder distributions were made by blending the three mono-modal distributions at equal weight ratios. The samples were manufactured in accordance with Wheat et al. [1] Parts were sintered using in a densifying (maximum 1400 °C) and non-densifying (maximum 1000 °C) sintering regime.
Data source location	Multi-Scale Additive Manufacturing Laboratory, University of Waterloo, Waterloo, ON, Canada.
Data accessibility	The analyzed data is available with this article. The raw data is available upon request.
Related research article	Wheat et al. [1] (in-press)

**Value of the data**

• The chemical composition and testing standards of the raw powder blends of 0–45 µm, 45–106 µm, 106–150 µm is presented.
• The CT part alignment, part isolation, and sensitivity analysis of the CT region of interest (ROI) is presented here.
• The visualization of the overall part density, particle and pore size of the for the three different powder types (Types A ○••, C ○•○, E ••○) manufactured using binder jetting additive manufacturing are useful in visualizing the effect of densifying (H) and non-densifying (L) sintering. For Type B ○○• and E •○•, the data is included in the work by Wheat et al. [1].

## 1 Data

Evaluation of the effects of the different sintering types was carried out using CT as described in Wheat et al. [1]. For this work, the CT analysis was performed on a single replicate of each of the powder Types A ○••, B ○○•, C ○•○, D •○• and E ••○ at 1400 °C (H) and 1000 °C (L) sintering regime respectively.

## 2 Experimental design, materials, and methods

The chemical composition of the three purchased mono-modal powders conforms to ASTM B348 for a Grade 1 CP titanium powder. The exact chemical composition for each material and the relevant testing standard for each element is listed in Table 1. The chemical testing was carried out by Luvak Inc. (Boylston, MA, USA) and the chemical information was provided by the powder supplier (Advanced Powders and Coatings).

**Table 1:** CP titanium powder chemical composition and testing standards used for analysis.

Content (weight %)					
Element	ASTM B348 grade 1	0–45 µm	45–106 µm	106–150 µm	Test Standard
Carbon	0.08 (max)	0.01	0.01	0.01	ASTM E1941
Oxygen	0.18 (max)	0.14	0.11	0.09	ASTM E1409
Nitrogen	0.03 (max)	0.01	0.01	< 0.01	ASTM E1409
Hydrogen	0.015 (max)	0.004	0.001	0.002	ASTM E1409
Iron	0.20 (max)	0.04	0.05	0.07	ASTM E2371
Other (individual)	0.1 (max)	< 0.1	< 0.1	< 0.1	ASTM E2371
Other (total)	0.4 (max)	< 0.4	< 0.4	< 0.4	ASTM E2371
Titanium	balance	balance	balance	balance	ASTM E2371

**Fig. 1:**
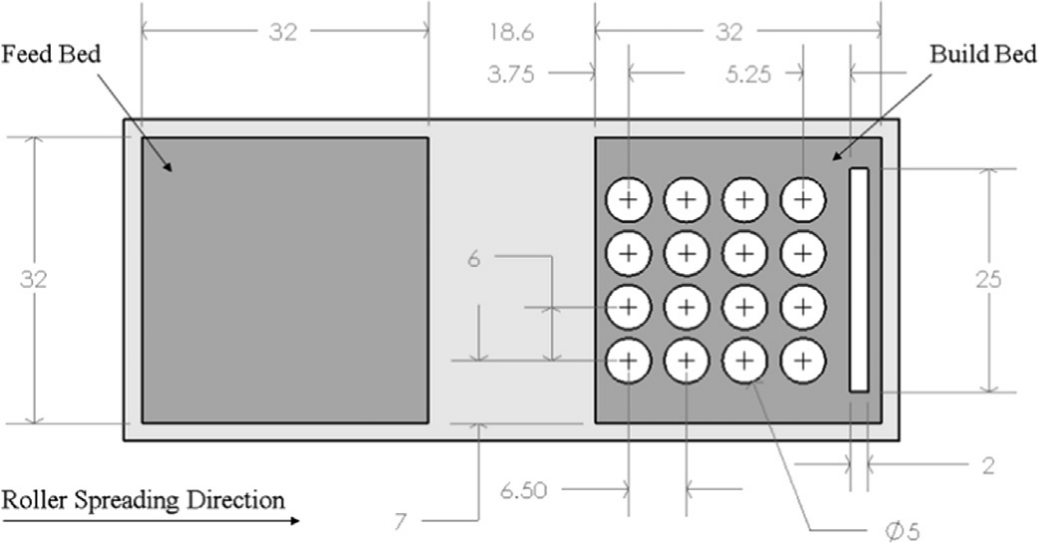
Build file dimensions showing the samples in the build bed on the right with the feed bed on the left.

The binder jetting samples were produced using modified Z-Corporation 310Plus (Z Corporation - acquired by 3D Systems, NC, USA). Inserts were made and installed to reduce the effective build bed and feed bed size of the system to 32 × 32 × 50 mm xyz respectively. The inserts and build file configuration is shown in Fig. 1 and the parts were manufactured in accordance with the method described by Wheat et al. [1].

**Fig. 2:**
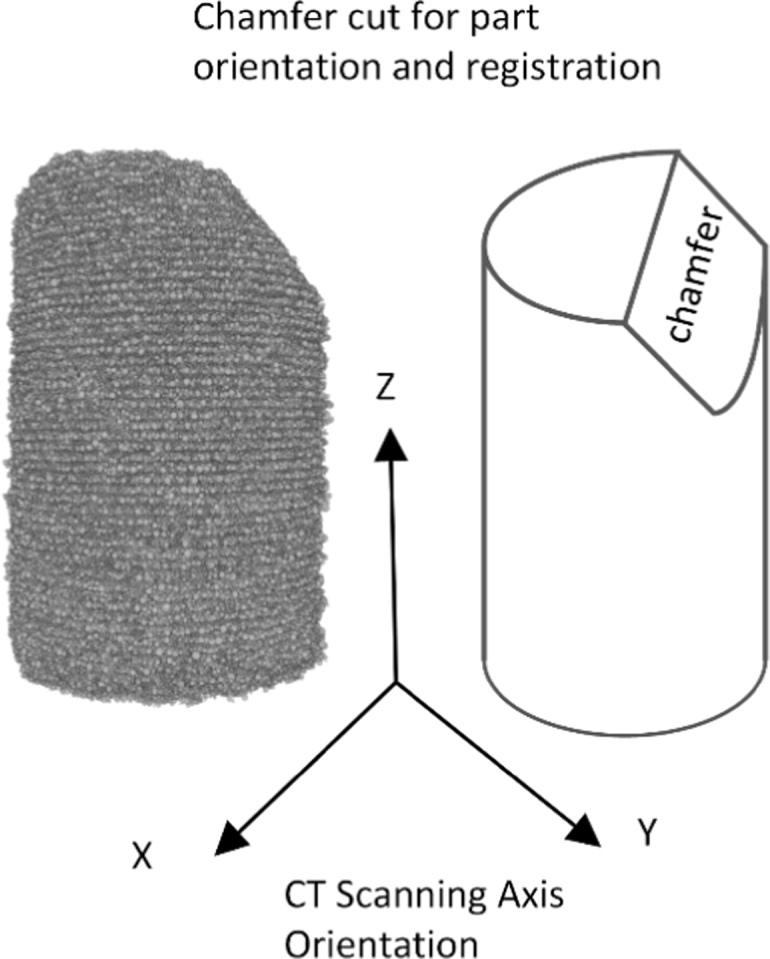
Orientation of the printed cylinders with respect to the CT scanner axes.

Evaluation of the effects of the different sintering types was carried out using computed tomography (CT). While other, more conventional metallurgical analysis methods, such as scanning electron or optical microscopy were available, they were deemed unsuitable. While these methods do provide useful data, they are limited to small regions of interest (ROI) and can only provide information on the outer, visible surface of the parts. Samples were scanned two at a time, with the samples stacked vertically in a sample holder. Scanning parameters are provided in the work by Wheat et al. [1]. The samples were spaced using paper since the material has a significantly lower attenuation, allowing both samples to be easily distinguished from each other. Samples also had a chamfer cut on the top surface to allow for consistent alignment of the part to the orthogonal axes after being scanned. The orientation of the scanner axes with respect to the printed parts is shown in Fig. 2, with the *Z* axis of the CT scanner corresponding to the build direction. Each of the reconstructed image sets was manually aligned with the orthogonal axes of the scanner.

**Fig. 3:**
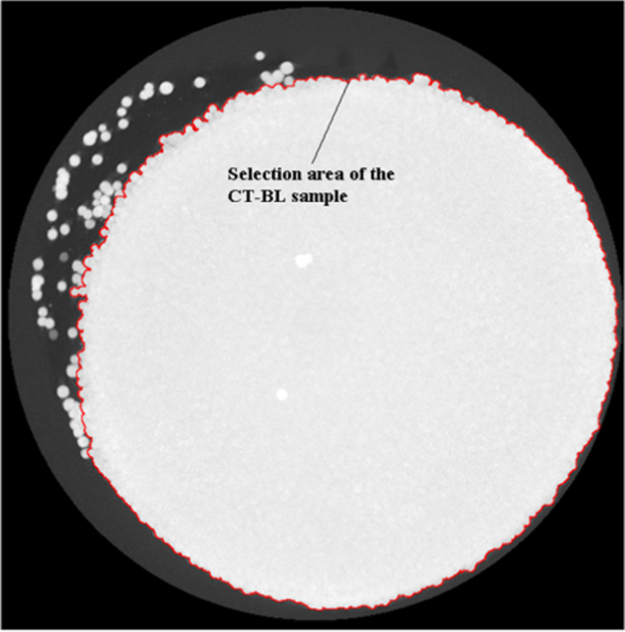
Maximum intensity projection image and resulting selection area (red boundary) of the CT-BL green part.

To isolate the parts from any loose powder particles inside the sample holder, a maximum intensity projection (MIP) of each image set was created. This projected the voxel with the highest attenuation value onto a single two-dimensional image for the entire image set. This allowed the outer profile of the sample to be determined, even though the samples were not perfectly aligned with the *Z* axis. The MIP, as well as the resulting selection area (red outer boundary), for the CT-BL green parts is shown in Fig. 3.

A single replicate was used for CT of each of the powder Types A ○••, B ○○•, C ○•○, D •○• and E ••○ at 1400 °C (H) and 1000 °C (L) sintering regime respectively. It was determined to be unfeasible to analyze the entirety of each sample, therefore, a 1.25 mm × 1.25 mm × 2.25 mm ROI was used for the green samples.

A representative volume analysis was performed in what was visibly determined to be the most heterogeneous dataset, CT-DH •○•, in terms of pore and solid configurations. ROIs were chosen according to the above description, and ranged in size from 0.35 × 0.35 × 2.25 mm^3^ to 1.75 × 1.75 × 2.25 mm^3^. ROIs were compared in terms of bulk relative density and mean pore size. To get an understanding of how well this centrally located ROI would represent the part, an internal ROI of this size, positioned near the top of the sample, was also analyzed.

Table 2 contains the results of all such ROI analysis for the purpose of demonstrating the density and pore size measurement sensitivity based on ROI selection. All centrally positioned ROIs, regardless of dimensions, had relative density values within 1% of the largest ROI tested. Mean pore diameter values for this set of ROIs also varied little around that of the largest ROI, staying within 1.7 µm. However, when moving the ROI towards the top of the sample, an increase in relative density is seen, while the mean pore diameter remains within 1 µm.

**Table 2:** Results from representative volume analysis on sample CT-DH in the green state.

ROI name	Location	Dimensions	Rel. Density	Mean Pore Diameter (µm)
CT-DH ●○●_92 × 92 × 592	Central	0.35 × 0.35 × 2.25 mm^3^	53.3%	41.8
CT-DH ●○●_132 × 132 × 592	Central	0.50 × 0.50 × 2.25 mm^3^	54.2%	41.0
CT-DH ●○●_192 × 192 × 592	Central	0.73 × 0.73 × 2.25 mm^3^	53.1%	43.0
CT-DH ●○●_264 × 264 × 592	Central	1.00 × 1.00 × 2.25 mm^3^	53.1%	43.0
CT-DH ●○●_330 × 330 × 592	Central	1.25 × 1.25 × 2.25 mm^3^	52.8%	42.7
CT-DH ●○●_460 × 460 × 592	Central	1.75 × 1.75 × 2.25 mm^3^	53.8%	42.7
CT-DH ●○●_330 × 330 × 592	Top	1.25 × 1.25 × 2.25 mm^3^	57.9%	41.8

Based on the relative bulk density analysis presented by Wheat et al. [1], the samples were categorized into two groups based on sintering behavior, the first group having samples with powder Type A ○••, B ○○•, and C ○•○, and the second group with powders Type D •○•, and E ••○.

**Fig. 4:**
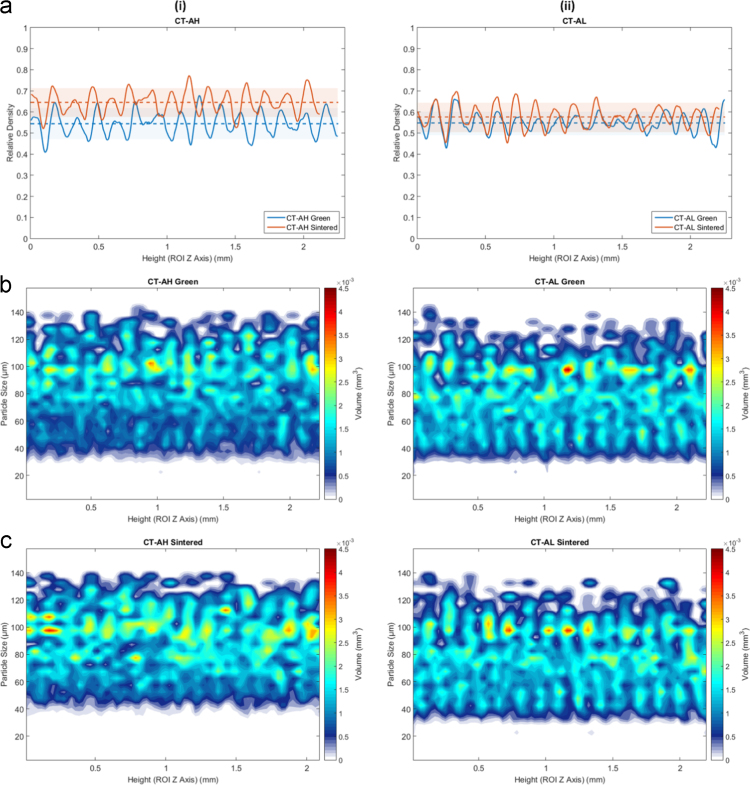
Density and particle size as a function of height for the Type A ○•• powder samples in the green and sintered state for densifying (H) and non-densifying (L) regimes. (a-i) relative density of the part before and after sintering for densifying (H) sintering and (a-ii) non-densifying sintering (H) respectively. (b-i, ii) and (c-i, cii) is the particle size (µm) and the corresponding volume fraction (mm^3^) belonging to each particle size per CT layer for the green and sintered states respectively.

**Fig. 5:**
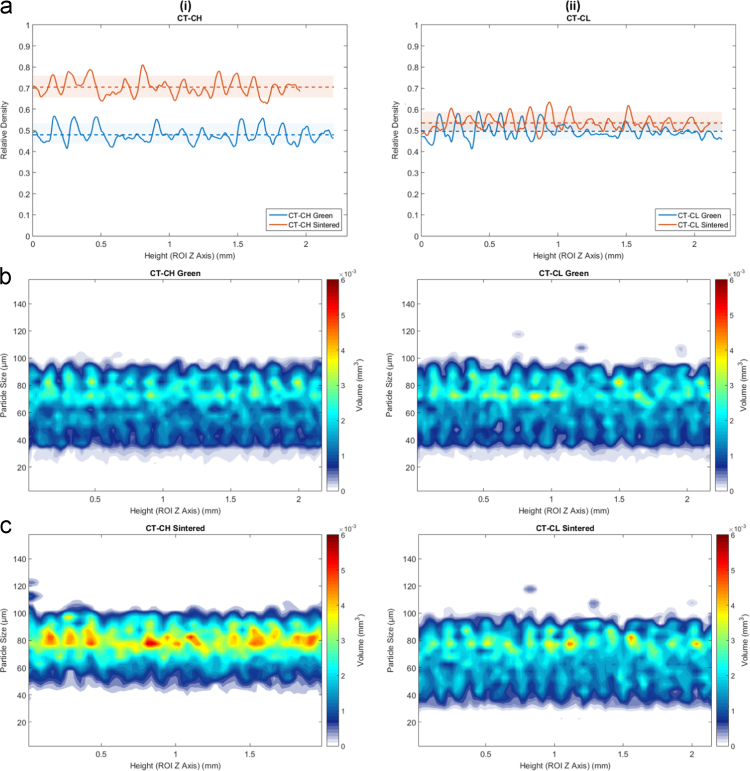
Density and particle size as a function of height for the Type C ○•○ powder samples in the green and sintered state for densifying (H) and non-densifying (L) regimes. (a-i) relative density of the part before and after sintering for densifying (H) sintering and (a-ii) non-densifying sintering (H) respectively. (b-i, ii) and (c-i, cii) is the particle size (µm) and the corresponding volume fraction (mm^3^) belonging to each particle size per CT layer for the green and sintered states respectively.

For the samples in the first group, the particle size distribution throughout the part is fairly consistent in both the green and sintered (for both densifying and non-densifying) states, as seen in Fig. 3 (Type A ○••) and Fig. 4 (C ○•○). Type B ○○• is presented in Wheat et al. [1]. The powder samples Type A ○•• shown in Fig. 4 and Type C ○•○ shown in Fig. 5 respectively, as well as in Type B ○○• is presented in Wheat et al. [1], both in the green and sintered (densifying and non-densifying) states, samples display some discernable periodic fluctuations in the particle size distribution that, similar to the part density, have a spatial period correlated to the layer thickness (150 µm).

**Fig. 6:**
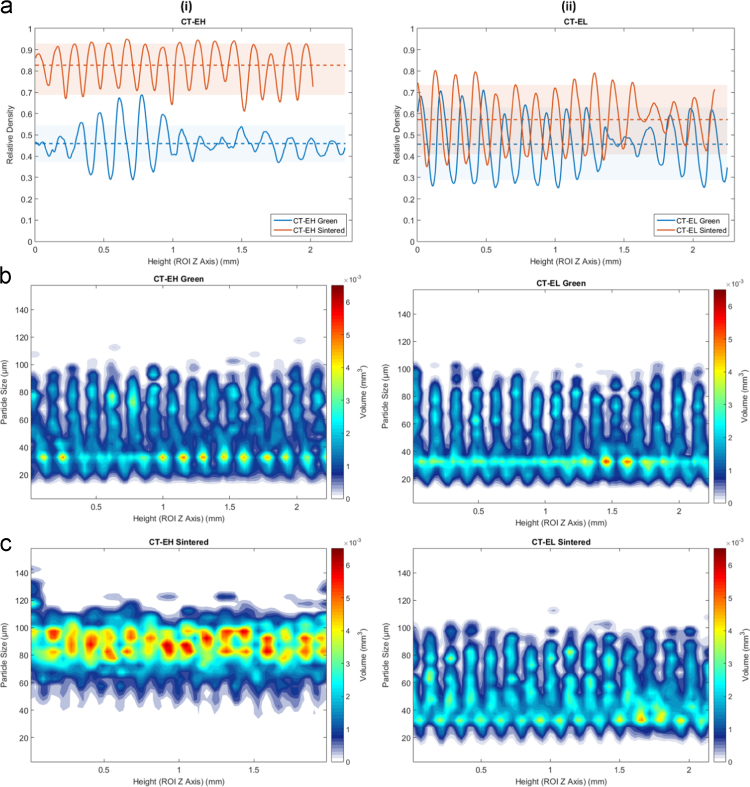
Density and particle size as a function of height for the Type E ••○ powder samples in the green and sintered state for densifying (H) and non-densifying (L) regimes. (a-i) relative density of the part before and after sintering for densifying (H) sintering and (a-ii) non-densifying sintering (H) respectively. (b-i, ii) and (c-i, cii) is the particle size (µm) and the corresponding volume fraction (mm^3^) belonging to each particle size per CT layer for the green and sintered states respectively.

For samples in the second group, manufactured using powders Type D •○•, and E ••○, the relative density and particle size map throughout the part is drastically different in the green versus sintered states for both densifying and non-densifying regimes, as seen in Fig. 6 for Type E ••○ samples. The figure for Type D •○• is presented in Wheat et al. [1]. As seen in Fig. 6, there are distinct periodic areas along the *Z* axis with a concentration of fine particles and almost a complete lack of the larger particles, followed by a segment of segregated large particles. This sequence of segregated zones of small and large particle agglomerations is present and consistent throughout the entire height of the part and will result in non-homogeneous parts after sintering.

**Fig. 7:**
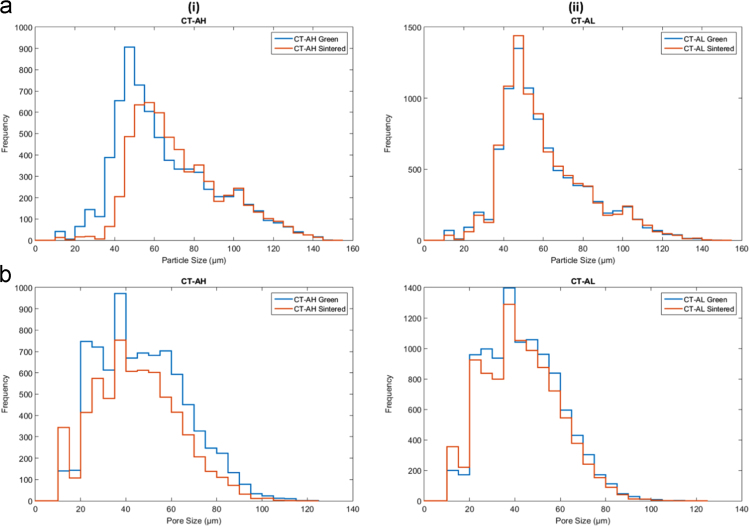
(a) Overall particle and (b) pore size histogram of the Type A ○•• powder samples in the green and sintered state for (i) densifying (H) and (ii) non-densifying (L) regimes.

**Fig. 8:**
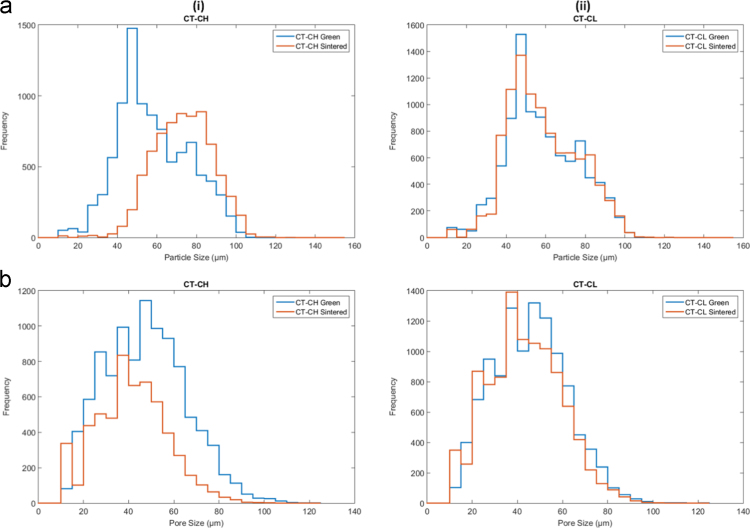
(a) Overall particle and (b) pore size histograms of the Type C ○•○ powder samples in the green and sintered state for (i) densifying (H) and (ii) non-densifying (L) regimes.

**Fig. 9:**
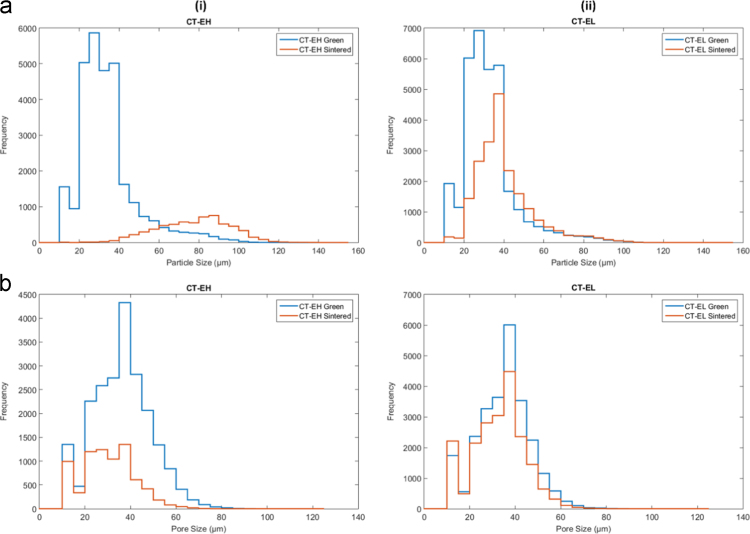
(a) overall particle and (b) pore size histograms of the Type E ••○ powder samples in the green and sintered state for (i) densifying (H) and (ii) non-densifying (L) regimes.

In this document, the analysis of the CT image sets describes the particle size and pore size. All values were found on a per-layer basis, with the average of those giving the overall value for the entire part. It was determined to be unfeasible to analyze the entirety of each sample so a 1.25 mm × 1.25 mm × 2.25 mm ROI was used for the green samples. Pore size was found by segmenting the 3D pore volume into individual pores respectively, using the watershed-based technique of pore network extraction, first described in [2]. The resulting networks contained pore diameter, volume, and position, as well as the diameter of constrictions (throats) between neighboring pores. Pore diameters were calculated as the maximal inscribed sphere, and throat diameters were calculated as the size of the largest sphere that could travel between neighboring pores. Particle size was found through effectively the same means as was used to find pore size, but with the reverse segmentation of the domain being used (highlighting the particles rather than the pores. The representative samples Type B ○○• and Type D •○• results are discussed in Wheat et al. [1]. The results for powder samples Type A ○•• are shown in Fig. 7, Type C ○•○ are shown in Fig. 8, and Type E ••○ are shown in Fig. 9.
