# Supplementary material for: Data related to the sinter structure analysis of titanium structures fabricated via binder jetting additive manufacturing
Source: Data Brief. 2018 Aug 31;20:1029–38. doi: 10.1016/j.dib.2018.08.135 (PMC6138942; doi:10.1016/j.dib.2018.08.135)
Supplement: Supplementary file 1 — Supporting information [file mmc1.docx]

**Conflict of interest**

The authors wish to declare that there are no conflicts of interest.
